# Reduced Sleep Quality Is Related to Poor Quality of Life in Patients with Juvenile Myoclonic Epilepsy, a Case-Control Study

**DOI:** 10.3390/life12030434

**Published:** 2022-03-16

**Authors:** Chih-Yin Lin, Tony Wu, Chun-Wei Chang, Hsiang-Yao Hsieh, Mei-Yun Cheng, Wei-En Johnny Tseng, Wey-Ran Lin, Cheng-Hong Toh, Yi-Ping Chao, Chun-Jing Liu, Siew-Na Lim

**Affiliations:** 1 Section of Epilepsy, Department of Neurology, Chang Gung Memorial Hospital at Linkou Medical Center and Chang Gung University College of Medicine, 5 Fu-Shin Street, Kwei-Shan, Taoyuan 333, Taiwan; cathycylin48@gmail.com (C.-Y.L.); tonywu@cgmh.org.tw (T.W.); franciscwchang@gmail.com (C.-W.C.); timshea@adm.cgmh.org.tw (H.-Y.H.); mr5894@adm.cgmh.org.tw (M.-Y.C.); b101090151@tmu.edu.tw (W.-E.J.T.); yiping@mail.cgu.edu.tw (Y.-P.C.); cmrpg3c1121@cgmh.org.tw (C.-J.L.); 2 Department of Neurology, New Taipei Municipal TuCheng Hospital (Built and Operated by Chang Gung Medical Foundation), New Taipei City 236, Taiwan; 3 Department of Neurology, Xiamen Chang Gung Hospital, Xiamen 361000, China; 4 Ph.D. Program in Biomedical Engineering, Chang Gung University, Taoyuan 333, Taiwan; 5 Department of Gastroenterology and Hepatology, Chang Gung Memorial Hospital at Linkou Medical Center and Chang Gung University College of Medicine, 5 Fu-Shin Street, Kwei-Shan, Taoyuan 333, Taiwan; t12350@cgmh.org.tw; 6 Department of Medical Imaging and Intervention, Chang Gung Memorial Hospital at Linkou Medical Center and Chang Gung University College of Medicine, 5 Fu-Shin Street, Kwei-Shan, Taoyuan 333, Taiwan; eldomtoh@cgmh.org.tw; 7 Department of Computer Science and Information Engineering, Chang Gung University, Taoyuan 333, Taiwan

**Keywords:** juvenile myoclonic epilepsy, sleep questionnaire, excessive daytime sleepiness, quality of life

## Abstract

Juvenile myoclonic epilepsy (JME) is a primary generalized epilepsy which is closely related to the sleep-wake cycle. This study aimed to investigate whether sleep disturbance is more common among patients with JME and the impact this may have on their quality of life (QOL). Thirty-four patients with JME and age- and gender-matched controls were recruited into this case control study, and assessed using validated sleep questionnaires including the Epworth Sleepiness Scale (ESS), Pittsburgh Sleep Quality Index (PSQI), and Stanford Sleepiness Scale (SSS). QOL was assessed using the Quality of Life in Epilepsy Inventory (QOLIE-31). The patients had a significantly higher PSQI score and higher proportion of abnormal PSQI scores than the controls. They also had higher ESS and SSS scores, but without statistical significance. The patients with poor sleep quality had significantly lower overall QOL, emotional well-being, and energy/fatigue subscale scores. The use of a higher number of antiseizure medications, dosage of levetiracetam, and usage of antiseizure medication polytherapy were associated with sleep disorders. Our results showed that sleep disturbance is common in patients with JME, and also that it has an impact on their QOL.

## 1. Introduction

There are reciprocal effects between sleep and epilepsy. Seizures have a tendency to present exclusively or predominantly during sleep, or upon wakening in some specific epileptic syndromes [1,2]. Sleep deprivation and sleep-wake transition are considered to increase the risk of seizures [3]. Moreover, variations in inter-ictal epileptic discharge (IED) frequency have been observed in different sleep stages, and IEDs are generally activated during non-rapid eye movement (NREM) sleep and partially inhibited during rapid-eye movement (REM) sleep [4]. On the other hand, epileptic activity has been shown to substantially modify sleep architecture by increasing sleep latency, fragmenting sleep, increasing awakening and stage shifts, and decreasing total sleep time and sleep efficiency [5]. In addition, anti-seizure medications (ASMs) can also affect sleep architecture in different ways [6].

Previous studies have shown that sleep disorders are more common in patients with epilepsy [7,8]. In addition, sleep disturbance is known to have an impact on poor seizure control and to subsequent affect daily functioning, overall cognitive performance, and feeling of well-being. Furthermore, recent studies have reported a negative correlation between poor sleep quality and a low Quality of Life in Epilepsy Inventory-31 (QOLIE-31) score in patients with epilepsy [8,9,10].

Juvenile myoclonic epilepsy (JME) is a primary generalized epilepsy characterized by myoclonic seizures alone or combined with generalized tonic-clonic seizures (GTCS) or absence seizures. JME affects approximately 7% of adolescents and adult patients with epilepsy, with a typical age at onset between 12 and 18 years. Seizures can be provoked by sleep deprivation and photostimulation, and predominantly occur after awakening [11]. In addition, JME is closely related to the sleep-wake cycle, particularly with transition phases (awakening, falling asleep, afternoon relaxation after work). Although sleep is known to be an important protective factor for JME, characteristic IEDs of 4–6 Hz spike-and-wave complex are often detected during NREM sleep [12].

The effect of sleep disturbances on the quality of life (QOL) in patients with JME is not clear. Therefore, the aims of this study were to investigate whether poor sleep quality and excessive daytime sleepiness are common in patients with JME, and to determine their potential impact on QOL.

## 2. Materials and Methods

### 2.1. Subjects

This case-control study was conducted at the Epilepsy Center of Linkou Chang Gung Memorial Hospital, a tertiary epilepsy center in Taiwan. Thirty-four patients fulfilled the diagnostic criteria of JME according to the International League Against Epilepsy (ILAE) classification [13] and consensus from international experts on JME [14,15]. Thirty age- and gender-matched healthy controls who did not have a history of neurological or psychiatric disorders and were not taking any medications were also recruited. The study protocol was approved by the hospital’s Institutional Review Board, and all procedures were in accordance with the Helsinki Declaration of 1975, as revised in 2008. Written informed consent was obtained from all participants.

### 2.2. Demographic and Clinical Data Collection

All of the participants underwent a structured uniform evaluation that included informant reports of medical history and a neurological examination. Demographic data including seizure types, age at onset of each seizure type, provoking factors, duration of epilepsy, detailed medical history, family history (epilepsy in first-degree relatives), ASM history, results of physical and brain imaging examinations, and seizure outcomes were collected. Seizure frequency was determined during routine clinic visits according to a seizure diary kept by the patients.

### 2.3. Sleep Evaluation

Daytime sleepiness and nighttime sleep quality were assessed using the Epworth Sleepiness Scale (ESS) [16] and Pittsburgh Sleep Quality Index (PSQI) [17], respectively. The ESS has a maximum score of 24, and scores ≥ 10 indicate excessive daytime sleepiness (EDS) [18]. The maximum PSQI score is 21, and scores ≥ 5 are indicative of poor sleep quality [17]. The Stanford Sleepiness Scale (SSS) was used to quantify subjective sleepiness levels at the time of evaluation. In the SSS, participants select one of seven options to identify their current level of sleepiness, and a score ≥ 3 is associated with a decline in performance that is related to sleepiness [19].

### 2.4. Quality of Life in Epilepsy

We used the QOLIE-31 [20] to assess the health-related QOL of the patients with JME. The questionnaire consists of 31 items, comprising seven subscales covering general and epilepsy-specific domains. The overall score and subscale scores range from 0 to 100, and a higher total score indicates a better QOL in patients with epilepsy [20,21].

### 2.5. Video-Electroencephalography Recording

The patients underwent 3 h of continuous video-electroencephalography (EEG) monitoring on the same day as they completed the aforementioned questionnaires. EEG signals were obtained using a digital VEEG system (BMSI 6000, Nicolet Biomedical, Inc., Madison, WI, USA; Nicolet vEEG, CareFusion Corporation, Middleton, WI, USA) with 19-channel EEG recording according to the international 10–20 system. Activation procedures including hyperventilation and photic stimulation were used to increase the diagnostic yield of the test.

### 2.6. Statistical Analysis

For between-group comparisons, the chi-square test was used for qualitative parameters, and the independent t-test was used for quantitative parameters. Patients with and without sleep disturbance were also compared. Multiple regression analysis was used to evaluate correlations between demographics, clinical characteristics, sleep disturbance and QOLIE-31. SPSS software version 21 (SPSS, Chicago, IL, USA) was used for all statistical analyses. A *p* value < 0.05 was considered to be statistically significant.

## 3. Results

### 3.1. Patient Demographics and Deposition

Data from the 34 patients with JME (19 women) and 30 controls (15 women) were included in the analysis. The mean ages of the patients and healthy controls were 26.8 ± 8.7 years and 30.5 ± 6.2 years, respectively, which was comparable (*p* = 0.05). Baseline demographic and clinical characteristics are summarized in Table 1. The mean age at onset of epilepsy was 14.5 ± 3.4 years, and the mean duration of epilepsy was 12.3 ± 9.0 years. Fifteen patients had clinical seizures within 3 months prior to the questionnaire, and IEDs were found on 3-h video-EEG in 18 patients.

### 3.2. Sleep Questionnaire Assessment

The PSQI, ESS, and SSS scores of the patients and healthy controls were compared. The patients had a significantly higher PSQI score than the controls (6.5 ± 3.5 vs. 4.3 ± 1.8, *p* = 0.003). In addition, more of the patients had a PSQI score ≥ 5 than the controls (61.8% vs. 33.3%, *p* = 0.023). Furthermore, more of the patients also had a higher ESS score and an abnormal ESS score (≥ 10) compared to the controls, although this did not reach statistical significance. Similarly, more of the patients had a higher SSS score and an SSS score ≥ 3 compared to the controls, although again this did not reach statistical significance. Overall scale scores for the self-assessment instruments are summarized in Table 2.

### 3.3. QOLIE-31 Scores

The mean overall QOLIE-31 score of the patients was 60.64 ± 18.9. The subscale score for social function was the highest (75.35 ± 20.3), followed by medical effects (70.83 ± 19.9), and the score for seizure worry (39.29 ± 27.9) was the lowest. A summary of the findings is presented in Table 3.

### 3.4. Relationship between Sleep Disorder and Quality of Life in the Patients

The patients with poor night sleep quality (PSQI score ≥ 5) had a trend towards a lower total QOLIE-31 score compared with the controls (57.7 ± 16.3 vs. 65.5 ± 9.8; *p* = 0.091). Three subscales of the QOLIE-31 (overall QOL, emotional well-being and energy/fatigue) were statistically significantly lower in those with a PSQI score ≥ 5 than in those with a PSQI score < 5. The subscale scores indicated that the patients with poorer night sleep quality had a lower QOL than those with normal sleep quality. In addition, the patients with excess daytime sleepiness (ESS score ≥ 10 or SSS score ≥ 3) had a lower total score and lower scores in all domains of the QOLIE-31, although without statistical significance. A summary of the findings is presented in Table 4.

### 3.5. Risk Factors for Poor Sleep Quality in the Patients

There were no significant correlations between PSQI/ESS scores and clinical characteristics including age, duration of epilepsy, and dosage of levetiracetam (LEV). There was a significant positive correlation between ESS score and the number of current ASMs (*p* = 0.019) (Table 5). In the patients with excess daytime sleepiness (ESS score ≥ 10), the number of current ASMs and proportion of those receiving ASM polytherapy was significantly higher compared to those without excess daytime sleepiness (2.30 ± 1.1 vs. 1.38 ± 0.7, *p* = 0.026; and 8 in 10 {80.0%} vs. 8 in 24 {33.3%}, *p* = 0.013, respectively). Similarly, in the patients with an abnormal PSQI score, the proportion of those receiving ASM polytherapy and higher dosage of LEV (daily dose ≥ 1500 mg) was significantly higher (6 in 21 {28.5%} vs. 3 in 13 {23.1%}, *p* = 0.003; and 20 in 21 {95.2%} vs 9 in 13 {69.2%}, *p* = 0.003, respectively) than in those with a normal PSQI score.

## 4. Discussion

In the present study, we found that the patients with JME had worse sleep quality than the controls. However, no significant correlations were found in excessive daytime sleepiness and subjective sleepiness level. In addition, the patients with poor night sleep quality had a trend towards a lower total QOLIE-31 score, especially in the subscales of social function, medical effects and seizure worry.

In this case-control study, the mean ESS scores and prevalence of excess daytime sleepiness were comparable between the patients with JME and healthy controls. Similar results were reported by Saraswati et al., who found a higher ESS score and frequency of EDS in 40 JME patients compared to 40 controls, but without statistical significance [22]. However, some studies have reported a significantly higher ESS score and prevalence of ESS in patients with JME. In a study of 50 patients with JME and 50 healthy controls, Krishnan et al. reported that the patients had a significantly higher prevalence of excessive daytime somnolence compared to the control group (34% vs. 4%, *p* < 0.001), and that the patients also had a significantly higher ESS score (8.89 ± 4.44 vs. 4.36 ± 2.61, *p* < 0.001) [23]. In the present study, the patients with JME had poor night sleep quality as assessed using the PSQI. In addition, the patients had a significantly higher PSQI score and significantly more had an abnormal PSQI score compared to the healthy controls. Similar findings were reported in the previous studies on JME [22,23]. This could be attributed to the presence of nocturnal seizures or the use of anticonvulsants [24].

Despite the well-established association between sleep and JME, the pathophysiology of epilepsy on sleep has not been adequately studied. Previous studies based on polysomnography have reported altered sleep architecture in JME patients. A higher cyclic alternating pattern rate has also been reported, and this may promote sleep instability and further foster epileptic activity [12,25]. In another study of sleep-wake rhythm and personality profile, Pung et al. found that patients with JME had a tendency to go to bed later at night, to get up later in the morning, and to feel fit at a later time during the day compared to patients with temporal lobe epilepsy [26].

In the present study, a higher proportion of the patients with sleep disturbance received polytherapy and a higher dosage of LEV. Most available studies of JME patients have focused on the effect of valproic acid (VPA) on sleep, and the results have been inconsistent. Studies regarding sleep architecture have reported that VPA was associated with no or little change in sleep architecture and stabilization of sleep [27,28]. Saraswati et al. found no significant difference between drug-naïve and VPA monotherapy groups in ESS and PSQI scores [22]. In addition, Krisnan et al. found no significant difference in the dosage and duration of VPA in patients with abnormal ESS and PSQI scores [23]. LEV is widely used as monotherapy for JME patients, since VPA, the usual first-line agent for JME, has chronic adverse events, especially in women of childbearing age [29]. Previous studies have suggested that LEV can increase sleep efficiency with no or little effect on sleep architecture [30]. Most of our patients (79.4%, 27/34) were on LEV (either mono- or polytherapy), and it is unclear whether LEV contributed to sleep disturbance in the study group. However, parameters regarding seizure control (proportion of IEDs on video-EEG and clinical attack in the past 3 months) were comparable between the patients on LEV and cohort group.

In this study, sleep disturbance (abnormal PSQI) was significantly associated with lower QOLIE-31 score with regards to overall QOL, emotional well-being and energy/fatigue. However, no significant association was observed in ESS. Our findings are not comparable with other studies which reported that sleep disturbance and daytime sleepiness had a significant impact on QOL. These discrepancies may be due to differences in the questionnaires applied in other studies. We suggest that it is crucial to recognize and treat sleep disturbance in order to improve the QOL of patients with JME.

There are several limitations to this study, including a relatively small number of patients. In addition, our patient group used different combinations of ASM therapy, and possible effects of the drugs on sleep could not be excluded since we lacked a drug-naïve group for comparison. Another limitation is that our study lacked other evidence of sleep quality apart from the validated questionnaires, such as sleep diary data, wrist actigraphy monitoring, and polysomnography.

In conclusion, the results of this study add to the knowledge of the association between sleep disturbance and JME, and also the impact on QOL in patients with JME. To preserve the QOL of patients with JME, psychiatric and sleep assessments may play an important role in treatment. Nevertheless, we suggest that further longitudinal studies involving a larger group of patients and objective sleep tests are warranted to better clarify these associations.

## Figures and Tables

**Table 1:** Baseline demographic and clinical characteristics.

Parameter	JME Patients	Control	*p* Value
	(*n* = 34)	(*n* = 30)	
Mean age (years)	26.8 ± 8.7	30.5 ± 6.2	0.05
Female, *n* (%)	19 (55.9%)	15 (50%)	0.64
Seizure onset age	14.5 ± 3.4	-	-
Duration of JME	12.3 ± 9.0	-	-
Number of current ASMs	1.6 ± 0.9	-	-
ASM polytherapy, *n* (%)	15 (44.1%)	-	-
Epileptiform EEG, *n* (%)	18 (52.9%)	-	-
Attack in the recent 3 months	15 (44.1%)	-	-

JME, juvenile myoclonic epilepsy; ASM, anti-seizure medication; EEG, electroencephalography.

**Table 2:** Comparisons of ESS, PSQI and SSS scores between the JME patients and controls.

Parameter	JME Patients	Controls	*p* Value
	(*n* = 34)	(*n* = 30)	
ESS, mean ± SD	6.9 ± 4.2	6.1 ± 3.1	0.396
ESS score ≥ 11, *n* (%)	7 (20.6%)	2 (6.7%)	0.109
PSQI, mean ± SD	6.5 ± 3.5	4.3 ± 1.8	0.003 *
PSQI ≥ 5, *n* (%)	21 (61.8%)	10 (33.3%)	0.023 *
SSS, mean ± SD	3.2 ± 1.5	2.8 ± 1.2	0.219
SSS ≥ 3, *n* (%)	22 (64.7%)	17 (56.7%)	0.511

JME, juvenile myoclonic epilepsy; ESS, Epworth Sleepiness Scale; PSQI, Pittsburgh Sleep Quality Index; SSS, Stanford Sleepiness Scale. * *p* < 0.05

**Table 3:** QOLIE-31 inventory scores of the patients with JME (*n* = 34).

Subscale	Patients	Total Score
	*n* (%)	Mean ± SD
Seizure worry		39.29 ± 27.9
Overall QOL		59.93 ± 12.7
Emotional well-being		57.0 6± 16.3
Energy/fatigue		50.59 ± 19.9
Cognitive function		61.20 ± 19.2
Medical effects		70.83 ± 19.9
Social function		75.35 ± 20.3
Overall score		60.64 ± 18.9
0–59	16 (47.1%)	
60–79	16 (47.1%)	
80–100	2 (5.8%)	

QOLIE-31, Quality of Life in Epilepsy-31.

**Table 4:** Mean QOLIE-31 subscale scores in relation to ESS and PSQI.

	ESS < 10		ESS ≥ 10		*p* Value	PSQI < 5		PSQI ≥ 5		*p* Value
	Mean	SD	Mean	SD		Mean	SD	Mean	SD	
Total score	54.72	11.73	45.34	15.61	0.11	65.46	9.79	57.65	16.34	0.091
Seizure worry	44.49	26.71	26.83	27.9	0.108	36.1	31.29	41.27	26.12	0.623
Overall QOL	62.08	11.1	54.75	15.3	0.193	67.12	9.18	55.48	12.69	0.004 *
Emotional well-being	58.33	15.73	54	18.21	0.521	66.77	9.58	51.05	16.92	0.002 *
Energy/fatigue	53.75	19.12	43	19.61	0.161	64.62	14.5	41.9	17.35	0.0003 *
Cognitive function	62.34	18.17	58.47	22.3	0.635	65.15	13.95	58.76	21.81	0.305
Medical effects	74.07	18.49	63.05	21.91	0.183	73.29	18.45	69.31	21.02	0.568
Social function	77.17	17.27	71	26.94	0.516	74.38	19.2	75.95	21.47	0.827

QOLIE-31, Quality of Life in Epilepsy-31; ESS, Epworth Sleepiness Scale; PSQI, Pittsburgh Sleep Quality Index. * *p* < 0.05.

**Table 5:** Multiple regression analysis between demographic and clinical characteristics and ESS and PSQI.

	ESS		PSQI	
	Beta	*p*	Beta	*p*
Age	−0.003	0.991	0.286	0.126
Duration of JME	−0.009	0.966	−0.205	0.25
Dosage of LEV	−0.001	0.441	0.001	0.176
Number of current ASMs	2.139	0.019	0.049	0.946

ESS, Epworth Sleepiness Scale; PSQI, Pittsburgh Sleep Quality Index; JME, juvenile myoclonic epilepsy; LEV, levetiracetam; ASM, anti-seizure medication.

## Data Availability

The datasets used and/or analysed during the current study are available from the corresponding author on reasonable request.
